# The impact of the Sudan armed conflict on Mycetoma control

**DOI:** 10.1371/journal.pntd.0011783

**Published:** 2023-12-07

**Authors:** Rawa Badri, Ahmed Hassan Fahal

**Affiliations:** 1 The Mycetoma Research Center, University of Khartoum, Khartoum, Sudan; 2 Faculty of Medicine, University of Khartoum, Khartoum, Sudan; Gulu University, UGANDA

The World Health Organization (WHO) has identified 20 neglected tropical diseases (NTDs), each of which could serve as an example of the definition of NTDs. Historically, these disfiguring, debilitating, and sometimes fatal diseases have been overlooked by policymakers and communities because they predominantly affect individuals with little political power and low voice. Despite being unique in their causes, epidemiology, and clinical presentation, they share the common characteristics of impacting impoverished communities and perpetuating poverty cycles. More than 1 billion people worldwide are affected by NTDs, resulting in devastating health, economic, and social consequences. While progress has been achieved since 2010, with 500 million individuals no longer demanding interventions against NTDs and 42 countries eradicating at least 1 NTD, the WHO road map for NTDs 2021–2030 outlines global targets and strategies for preventing, controlling, eliminating, and eradicating these diseases over the next decade [1].

Mycetoma exhibits all the NTD characteristics, recognised by WHO in 2016 as an NTD due to its debilitating complications [2]. Mycetoma typically appears as a painless lump with multiple sinuses that discharge grains encapsulating the causative microorganisms [2]. Mycetoma is primarily a localised infection and causes chronic destructive inflammation in the nearby subcutaneous tissue. However, it can also spread to other tissues and structures, including the skin, deep tissues, organs, and bones. Without proper treatment, it can cause significant tissue damage, disfigurement, and disability that can impede a patient’s ability to carry out their usual daily activities and can result in fatal outcomes [3]. This chronic disabling inflammatory disease is classified into eumycetoma, caused by fungi, and actinomycetoma, caused by filamentous bacteria [2]. Mycetoma is endemic in many countries, with actinomycetoma being more prevalent in the Americas and the Middle and Far East, while eumycetoma is more common in some central African countries. *M*. *mycetomatis* and Nocardia spp. are the main causative agents globally, with *M*. *mycetomatis* being the most common overall [4]. Mycetoma mainly affects young adult males, but females and other age groups are also affected. Individuals who work as farmers or shepherds and live in underprivileged communities with limited access to healthcare, education, and sanitation are at higher risk of contracting the disease in endemic areas [2]. The specific treatment approach depends on factors such as the causative organism of the infection, its location, and how severe it is. Unfortunately, many patients seek medical attention too late, resulting in advanced stages of the disease where amputation may be the only viable option. This is often due to a lack of education and awareness about the condition [5]. Mycetoma is endemic in arid tropical and subtropical regions with short rainy seasons and low humidity. The disease is prevalent in Africa, Asia, and Central and South America, with Sudan being one of the countries most affected. In Sudan, an estimated 63,825 individuals have suffered from mycetoma since 1991. The data also showed that the number of cases of eumycetoma exceeds 50 per 10,000 inhabitants, while that of actinomycetoma exceeds 5 per 10,000 inhabitants [2]. Mycetoma cases have been reported in non-endemic areas such as Canada, Russia, Germany, and Japan, possibly due to climate change and increased migration, tourism, and trade [4].

Before the current conflict in Sudan, the Mycetoma National Control Programme was halted for approximately 6 months due to the COVID-19 pandemic. The pandemic has severely impacted global health systems and economies, suspending various health services and programmes, including those for NTDs. This disruption has significantly impacted mycetoma and the other NTDs management and hindered the progress towards achieving the Sustainable Development Goals (SDGs) disease targets by 2030 [6].

According to the latest displacement matrix from the International Organization for Migration (IOM), the ongoing armed conflict that started on April 15, 2023, between the Sudanese army and paramilitaries has caused an enormous number of people to flee, with over 926,000 seeking refuge in other countries and 3.02 million displaced internally in over 100 days [7]. This massive internal displacement usually exacerbates the risk of contracting many infections, and mycetoma is not an exception, as many displaced people started to be involved in farming and animal breeding in mycetoma-endemic regions, thus exposing themselves to the disease [2,8]. In addition, this conflict has resulted in widespread devastation, including attacks on healthcare workers and infrastructure, with hospitals targeted by missile and artillery strikes and soldiers demanding treatment priority. As a result, healthcare accessibility has become severely limited, exacerbating the suffering of 45 million people affected by the conflict. In Khartoum, over 65% of hospitals have been destroyed or closed, endangering lives and limiting access to essential healthcare services [9]. Furthermore, the already high prevalence of diseases such as malaria, hepatitis, and dengue fever in Sudan has increased the demand for healthcare services, creating challenges for medical facilities to meet the needs of those injured during the current conflict, which led to less attention to mycetoma patients as most of the reported cases currently are dengue fever and cholera cases [10].

The Mycetoma Research Centre (MRC) in Khartoum, the only WHO collaborating center dealing with the various aspects of mycetoma management, has halted its operations due to the ongoing conflict in Sudan. As a result, thousands of patients have been left untreated since the war began. Although the MRC staff are in contact with affected individuals, they cannot provide them with free medications, leading to disease progression, complications, and severe socioeconomic impacts on patients and their families.

As mycetoma affects the poorest of the poor in poor, remote, impoverished regions, the MRC established 2 satellite centers in endemic villages to manage patients in their villages. In these centers, the diagnosis is confirmed, medical and surgical treatment is provided, health education events are organised, and these activities are delivered free of charge. These satellite centers reduced the treatment cost spared the patients and their families time, and field surveys were conducted for early case detection and management. However, these activities have been disrupted by ongoing violence in the country [11].

The MRC had established the Mycetoma Vocational and Entrepreneurship Training Center (SAA’ID). It is a vocational, business development, and entertainment hub to create an enabling environment for mycetoma amputees and disabled persons to learn and develop income-generating skills, perform and upgrade their talents, and be prepared to lead a new life of being productive, self-reliant, and confident. SAA’ID has various vocational training programmes such as Sudanese handcraft and leather industry, artisan work, painting, graphics, woodwork, etc. Furthermore, it qualifies trainees on business development skills to become professional entrepreneurs and ready to establish small developing enterprises. Now, the center is inaccessible and partially damaged.

## Recommendations

It is crucial to stop the ongoing conflict in Sudan immediately, as millions have been displaced, and the healthcare system has been severely disturbed. The international humanitarian laws to safeguard civilians and infrastructure must be respected. The Ministry of Health in Sudan and its partners must have an action plan over the coming weeks and months that should include conducting in-depth health assessments, restoring the functionality of health facilities in affected areas, and establishing fixed and mobile health clinics as close as possible to affected populations. It is critical to restore and maintain essential healthcare services, which include providing necessary supplies to healthcare facilities, ensuring treatment for infectious and chronic diseases, and resuming immunisations and other routine health programmes [12]. Furthermore, healthcare professionals are at the forefront of this emergency amidst hazardous circumstances, risking their lives to provide assistance. They must be protected [12,13].

International cooperation is essential to address the Sudan crisis and support the Sudanese healthcare system during and after the conflict. While some organisations have contributed to the crisis relief, further efforts are required. Urgent and adequate funding, improved security measures for healthcare facilities and workers, and better access to medical resources are needed to alleviate the conflict effects and offer vital healthcare services to those affected [12]. Additionally, mental health services must be integrated into the healthcare response, recognising the significant psychological impact of conflicts on individuals, especially those suffering from chronic debilitating conditions such as mycetoma [12,14].

## Conclusions

The current ongoing armed conflict that started on April 15, 2023, has severely affected Mycetoma control programmes in Sudan, thus delaying the advancement towards attaining the SDGs disease targets by 2030. Mycetoma patients are from underprivileged communities with limited access to the free healthcare services that used to be provided by the Mycetoma Research Centre, and conflict has ended their opportunities to get these necessary healthcare services. International community support and assistance are badly needed to restore the Mycetoma control programme activities to alleviate the patients’ suffering and reduce the disease burden on patients, their families, and communities in endemic regions in Sudan.
